# Spectrofluorimetric Determination of Human Serum Albumin Using Terbium-Danofloxacin Probe

**DOI:** 10.1100/2012/940541

**Published:** 2012-05-02

**Authors:** Amir M. Ramezani, Jamshid L. Manzoori, Mohammad Amjadi, Abolghasem Jouyban

**Affiliations:** ^1^Department of Analytical Chemistry, Faculty of Chemistry, University of Tabriz, Tabriz, Iran; ^2^Drug Applied Research Center and Faculty of Pharmacy, Tabriz University of Medical Sciences, Tabriz 51664, Iran

## Abstract

A spectrofluorimetric method is proposed for the determination of human serum albumin (HSA) and bovine serum albumin (BSA) using terbium-danofloxacin (Tb^3+^-Dano) as a fluorescent probe. These proteins remarkably enhance the fluorescence intensity of the Tb^3+^-Dano complex at 545 nm, and the enhanced fluorescence intensity of Tb^3+^-Dano is proportional to the concentration of proteins (HSA and BSA). Optimum conditions for the determination of HSA were investigated and found that the maximum response was observed at: pH = 7.8, [Tb^3+^] = 8.5 × 10^−5^ mol L^−1^, [Dano] = 1.5 × 10^−4^ mol L^−1^. The calibration graphs for standard solutions of BSA, HSA, and plasma samples of HSA were linear in the range of 0.2 × 10^−6^ − 1.3 × 10^−6^ mol L^−1^, 0.2 × 10^−6^ − 1.4 × 10^−6^ mol L^−1^, and 0.2 × 10^−6^ − 1 × 10^−6^ mol L^−1^, respectively. The detection limits (S/N = 3) for BSA, HSA, and plasma sample of HSA were 8.7 × 10^−8^ mol L^−1^, 6.2 × 10^−8^ mol L^−1^, and 8.1 × 10^−8^ mol L^−1^, respectively. The applicability of the method was checked using a number of real biological plasma samples and was compared with the UV spectrometric reference method. The results was showed that the method could be regarded as a simple, practical, and sensitive alternative method for determination of albumin in biological samples.

## 1. Introduction

The development of novel methods and new techniques for protein determination is very important in a number of areas such as chemical and biochemical analyses, immunodiagnostics, and biotechnology. There is a connection between the content of human serum albumin (HSA) in plasma and some diseases, such as kidney [1] or chronic liver diseases [2] and unstable angina [3] in addition the enhancement of albumin in cerebrospinal fluid (CSF) samples of amyotrophic lateral sclerosis (ALS) patients was reported [4–7]. Application of albumin level in body fluids as a biomarker of these diseases has been suggested, and development of simple and precise methods which could be applicable in routine bioanalytical methods is needed. 

According to the above-mentioned facts, determination of HSA is an important issue in clinical diagnosis. Unfortunately, the traditional methods for the determination of proteins have some disadvantages. For example, Lowry [8] assay can be used only for protein concentration >10 mg mL^−1^. Bradford [9] assay has the drawback of contaminating vessels. The silver staining method [10] is complicated as multiple steps are involved. So the chemiluminescent analysis [11], fluorimetric analysis, and resonance light scattering [12–14] methods have been proposed due to their high sensitivity and selectivity. Among them, fluorescence method stands out since its obvious sensitivity is some orders of magnitude higher than that of other spectral detections, especially when the method was incorporated into high-performance liquid chromatography (HPLC) and capillary electrophoresis (CE) [15]. It is well known that only proteins possessing phenylalanine, or tryptophan, or a combination there of, exhibit natural fluorescence. But the resulted emission is too weak to be applied for the analysis of proteins (e.g., albumin) at low concentration. In order to solve this problem, protein can be converted into suitable derivatives by chemical derivatization, typically using a derivatization reaction or spectral probes. There are many publications reporting the use of derivatization methods for improving proteins detectability [16–18]. Up to now, a number of fluorescence methods for the determination of proteins have been established based on extrinsic fluorescence probe such as rare earth ions and complexes, or dyes [19–24]. In this work, for the first time, we employ the fluorescence of terbium-danofloxacin (Tb^3+^-Dano) complex as a probe for the determination of albumin. The proposed method was applied to the determination of HSA in human plasma samples and the results were in good agreement with the results obtained by the standard UV-Vis spectrophotometer method [25].

## 2. Experimental

### 2.1. Materials and Methods

Analytical-grade ethanol, hydrochloric acid (HCl), methanol, 2-propanol, acetonitrile, and tris-(hydroxymethyl) aminomethane (Tris) were obtained from Merck (Darmstadt, Germany), terbium(III) chloride hexahydrate (TbCl_3_·6H_2_O) from Acros Organics (Geel, Belgium), Dano powder from Jamedat Afagh Pharmaceutical Company (Tehran, Iran), proteins (HSA and BSA) standard solution (1 × 10^−4^ g dL^−1^), and spectrophotometric detection kit from Pars Analysis company (Tehran, Iran). Double-distilled water prepared using the Millipore-Q-plus water purification system (Millipore, Bedford, MA, USA) was used in this study.

A 1.0 × 10^−2^ mol L^−1^ terbium (III) solution was prepared by dissolving the appropriate amount of TbCl_3_·6H_2_O in double-distilled water and stored in a polyethylene container to avoid memory effects of terbium adsorbed on glass vessels. A stock solution (1.0 × 10^−2^ mol L^−1^) of Dano was prepared in double-distilled water, and 0.1 mol L^−1^ Tris-hydrochloric acid (Tris-HCl) buffer solution was prepared by dissolving the desired amount of Tris-base in 100 mL of water, adjusting the pH to 7.8 with HCl.

### 2.2. Apparatus

Fluorescence spectra and intensity measurements were performed on a Jasco FP-750 spectrofluorimeter (Kyoto, Japan) equipped with a 150 W xenon lamp, using a 10 mm quartz cell 5 nm. The excitation wavelength was set at 347 nm, and the fluorescence intensity was measured at 545 nm. All measurements were performed at 25°C, controlled using a Peltier thermostated cell holder (Jasco, Japan). The pH of solutions was measured with Metrohm 654 pH meter (Herisau, Switzerland).

UV-Vis spectrophotometer (Beckman DU-650, Fullerton) was used for determination of albumin by a detection kit.

### 2.3. Sample Preparation

#### 2.3.1. Standard Sample

The working solutions of HSA and BSA (1 × 10^−5^ mol L^−1^) were obtained by appropriate dilutions of the standard solutions (1 × 10^−4^ g dL^−1^) with double-distilled water. A 8.5 × 10^−4^ mol L^−1^ of terbium solution and 1.5 × 10^−3^ mol L^−1^ of Dano solution were prepared from the stock solutions.

#### 2.3.2. Plasma Sample Preparation

The developed method was applied to the determination and quantification of HSA in plasma samples. As the main proportion of plasma protein content is albumin (about 60%) [26], the resulted intensities can be regarded due to the albumin content of plasma rather than other rare proteins. For the assay of HSA, the samples must be diluted appropriately within the linear range of the determination of HSA.

### 2.4. Experimental Procedure

#### 2.4.1. Fluorescence Assay

For the analysis of albumin (HSA and BSA) in different samples, solutions were added in the following order: 1 mL of 8.5 × 10^−4^ mol L^−1^ Tb^3+^ solution, 1 mL of 1.5 × 10^−3^ mol L^−1^ Dano solution, suitable aliquots of protein solution, and 0.4 mL buffer 0.1 mol L^−1^ (pH = 7.8) into 10 mL calibrated flasks. The mixture was diluted up to 10 mL with double-distilled water and allowed to stand for 5 min at room temperature. The fluorescence intensity was measured at *λ*ex/*λ*em = 347 nm/545 nm. The enhanced fluorescence intensity of Tb^3+^-Dano by HSA was represented as ΔF% = 100 (F − F_0_)/F_0_ where F and F_0_ are the fluorescence intensities of the systems with HSA and without HSA, respectively.

#### 2.4.2. Standard UV-Vis Spectrophotometry Assay

For the analysis of HSA by standard UV spectrophotometry the following procedure was used: to preparation of blank, standard, and sample solutions, 10 *μ*L double-distilled water, 10 *μ*L standard solution, and 10 *μ*L sample were added into 2 mL micro tubes containing 1 mL reagents, respectively.

After complete mixing, the solution should be incubated for 10 min in 37°C. The absorbance of the standard and sample solution should be measured against blank solution at *λ* = 546 nm in 60 min. the concentration is calculated using following formula:

(1) $$\begin{array}{c} \text{Albumin}\;\left(\text{mol}\;{\text{L}}^{-1}\right)=\frac{\text{Sample}\;\text{Absorbance}\times \text{Standard}\;\text{Concentration}\;\left(\text{mol}\;{\text{L}}^{-1}\right)}{\text{Standard}\;\text{Absorbance}}. \end{array}$$

  

## 3. Results and Discussion

### 3.1. Fluorescence Spectra

**Figure 1:**
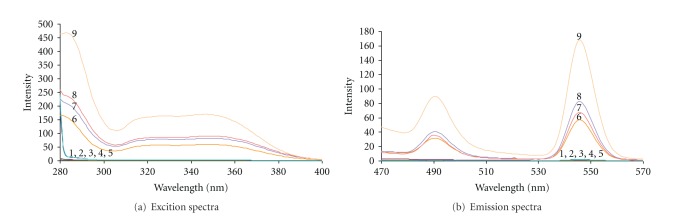
Excitation and emission spectra. (a): Excitation spectra (b): Emission spectra. (1) Tb^3+^, (2) BSA, (3) HSA, (4) Tb^3+^-BSA, (5) Tb^3+^-HSA, (6) Tb^3+^-Dano, (7) Tb^3+^-Dano-BSA, (8) Tb^3+^-Dano-HSA, (9) Tb^3+^-Dano-HSA (plasma sample). Analytical conditions: [Tb^3+^] = 8.5 × 10^−5^ mol L^−1^, [Dano] =1.5 × 10^−4^ mol L^−1^, [BSA] = 1 × 10^−6^ mol L^−1^, [HSA] = 1 × 10^−6^ mol L^−1^, [HSA in plasma sample] = 1 × 10^−6^ mol L^−1^, pH = 7.8, *λ*ex/*λ*em = 347 nm/545 nm.

Fluorescence emission and excitation spectra of Tb^3+^, BSA, HSA, Tb^3+^-BSA, Tb^3+^-HSA, Tb^3+^-Dano, Tb^3+^-Dano-BSA, Tb^3+^-Dano-HSA, and Tb^3+^-Dano-HSA (plasma sample) are shown in Figure 1. Tb^3+^ solution does not show the characteristic fluorescence spectrum, while by adding Dano to Tb^3+^ solution, intense fluorescence was observed. The maximal excitation wavelength of Tb^3+^-Dano occurs at 347 nm and two emission peaks at 490 and 545 nm, corresponding to the ^5^D_4_-^7^F_6_ transition and ^5^D_4_-^7^F_5_ transition of Tb^3+^, respectively. The fluorescence intensity at 545 nm is the greatest. Therefore, the excitation and emission peaks were set at 347 and 545 nm, respectively. The fluorescence spectrum of the Tb^3+^-Dano-HSA system was similar to that of Tb^3+^-Dano; however, the fluorescence intensity of Tb^3+^-Dano was enhanced by proteins (HSA and BSA), and the complementary experiments showed that the enhancement was proportional to the concentration of the proteins.

### 3.2. Optimization of Experimental Conditions

#### 3.2.1. Effect of pH

**Figure 2:**
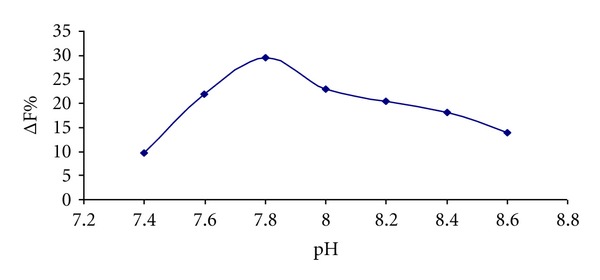
Effect of pH on the enhanced fluorescence intensity (ΔF%) analytical conditions: [Tb^3+^]: 1 × 10^−4^ mol L^−1^; [Dano]: 1 × 10^−4^ mol L^−1^; [HSA]: 1 × 10^−6^ mol L^−1^.

Fluorescence intensities of series of 0.1 mol L^−1^ Tris-HCl buffer solutions with the pH range of 7.4–8.6 were measured at *λ*ex/*λ*em = 347 nm/545 nm (Figure 2). The enhancement intensity (ΔF%) of Tb^3+^-Dano complex with albumin (HSA and BSA) is strongly dependent on pH and reaches to maximum value at pH 7.8. Thus, pH 7.8 (0.1 mol L^−1^ Tris buffer) was selected as optimum pH for the rest of analysis. Below this pH, the hydroxyl groups of Dano are probably in protonated form, which disfavors the complex formation. Also, Tb^3+^ ion would be precipitated in the strong alkaline medium, which blocked the coordination between the Dano and Tb^3+^ ion. 

#### 3.2.2. Effect of Concentration of Tris Buffer

**Figure 3:**
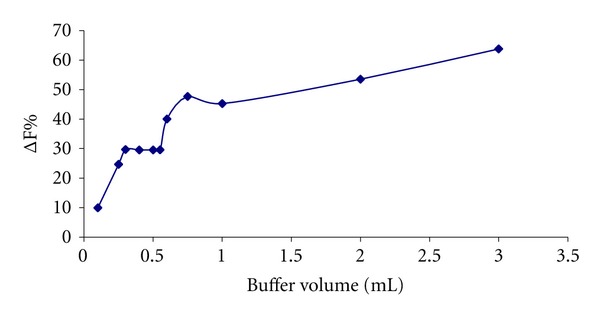
Effect of concentration of Tris buffer on the enhanced fluorescence intensity (ΔF%) analytical conditions: [Tb^3+^]: 1 × 10^−4^ mol L^−1^; [Dano]: 1 × 10^−4^ mol L^−1^; [HSA]: 1 × 10^−6^ mol L^−1^; pH = 7.8.

Tris buffer is known to have chelating properties with lanthanide ions. Hence, it is necessary to optimize its concentration that will afford maximum sensitization of the Tb^3+^-Dano-HSA system. The influence of Tris buffer concentrations on luminescence intensity of Tb^3+^ was studied (Figure 3) by varying the buffer volume of the buffer in the range of 0.1–3.0 mL of 0.1 mol L^−1^ buffer solution while the concentrations of Tb^3+^, Dano, and HSA were 1 × 10^−4^ mol L^−1^, 1 × 10^−4^ mol L^−1^, and 1 × 10^−6^ mol L^−1^, respectively. The coordination of Tb^3+^ ions by Tris prevents the OH groups of water molecules from surrounding the terbium ions and reduces the complexation of Dano. In lower concentrations of Tris, the buffer could not coordinate terbium ions completely, and the fluorescence intensity is decreased. The results indicated that the fluorescence of the probe in the presence of the analyte at buffer volumes between 0.3 mL–0.55 mL is constant, therefore 0.4 mL of 0.1 mol L^−1^ Tris buffer was selected as optimum buffer content.

#### 3.2.3. Effect of Tb^3+^ Concentration

**Figure 4:**
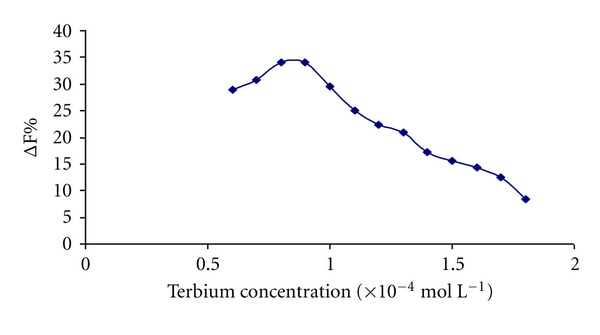
Effect of the Tb^3+^concentration on the enhanced fluorescence intensity (ΔF%)analytical conditions: [Dano]: 1 × 10^−4^ mol L^−1^; [HSA]: 1 × 10^−6^ mol L^−1^; pH = 7.8.

The effect of the Tb^3+^ concentration on the luminescence intensity enhancement (ΔF%) of Tb^3+^-Dano-HSA system was studied (Figure 4), at constant concentration of 1 × 10^−6^ mol L^−1^ HSA. The ΔF% was highest when the concentration of Tb^3+^ in the mixture was in the rang of 8 × 10^−5^ mol L^−1^–9 × 10^−5^ mol L^−1^. Intermediate Tb^3+^ concentration (8.5 × 10^−5^ mol L^−1^) was chosen for further analysis.

#### 3.2.4. Effect of the Dano Concentration

**Figure 5:**
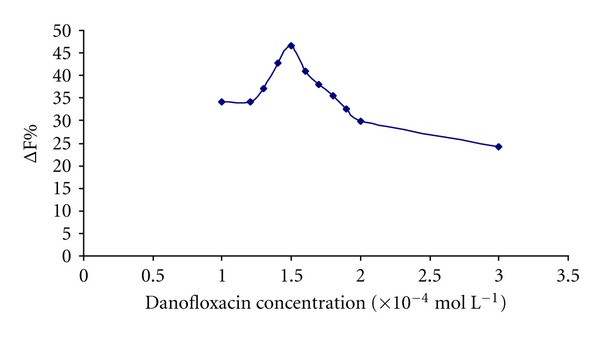
Effect of the amount of Dano on the enhanced fluorescence intensity (ΔF%) analytical conditions: [Tb^3+^]: 8.5 × 10^−5^ mol L^−1^; [HSA]: 1 × 10^−6^ mol L^−1^; pH = 7.8.

The influence of the Dano concentration on the fluorescence intensities was studied (Figure 5), and it was found that the fluorescence intensity enhancement of Tb^3+^-Dano-HSA system reached a maximum when the concentration of Dano was 1.5 × 10^−4^ mol L^−1^. So 1.5 × 10^−4^ mol L^−1^ was used as optimum concentration of Dano for further study.

#### 3.2.5. Effect of Reaction Time

Under the optimum conditions, the effect of reaction time on the fluorescence intensity was studied. It was found that the fluorescence intensity is stable for about 50 min after addition of all reagents. This is due to the rapid complex formation between Tb^3+^ and Dano and HSA. In this study, 5 min was set as the standard time interval for all fluorescence intensity measurements.

#### 3.2.6. Effect of Temperature

Temperature had significant influence on the fluorescence intensity of the system; at lower temperatures the enhancement fluorescence in the presence of HSA was decreased, therefore we selected room temperature (25°C) for further study.

#### 3.2.7. Effect of the Addition Order of Reagents

Series of solutions were prepared with different addition orders of reagents but the same concentrations of reagents (F), and their corresponding blank solutions (F_0_) were measured at *λ*ex/*λ*em = 347 nm/545 nm. The results showed that different orders of addition of components have significant impact on both F and ΔF% (Table 1). The highest enhancement in the intensity of the fluorescence probe in the presence of albumin was obtained for the addition of reagents in the order of Tb^3+^, Dano, HSA, and buffer.

#### 3.2.8. Surfactant Effect

In order to improve fluorescence intensity various surfactants such as SDS, SDBS, CTAB, Tween 20, Tween 80, Triton X-100, and Triton X-114 were added to the solution, and their effect was studied. None of the studied surfactants had significant effect on ΔF%.

#### 3.2.9. Solvent Effect

**Figure 6:**
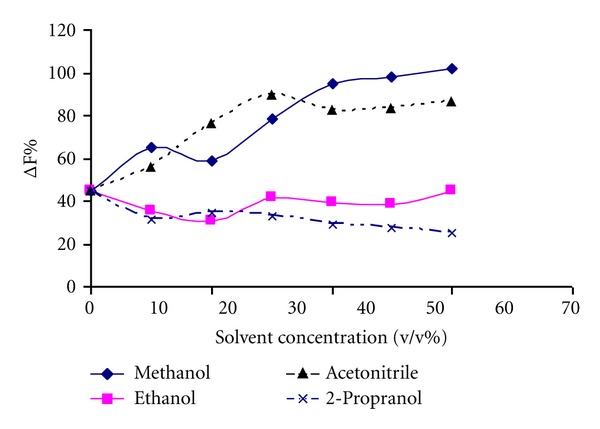
Solvent effect on the enhanced fluorescence intensity (ΔF%)Analytical conditions: [Tb^3+^]: 8.5 × 10^−5^ mol L^−1^; [Dano]: 1.5 × 10^−4^ mol L^−1^; [HSA]: 1 × 10^−6^ mol L^−1^; pH = 7.8.

The effect of different solvents on fluorescence intensity was investigated at optimum conditions. Although some solvents (e.g., methanol) improved the ΔF%, they decreased the fluorescence intensities of the probe in the presence or absence of HSA which can lead to the decreased repeatability. In addition to the decreased repeatability and the nontoxicity of water, it was chosen for further study (Figure 6).

### 3.3. Interference Studies

The interference of various exogenous substances was tested and shown in Table 2. It was found that except ctDNA, other substances had little effect on the determination of HSA: 1 × 10^−6^ mol L^−1^ within the acceptable error range (±5%).

### 3.4. Analytical Applications and Assay Validation

#### 3.4.1. The Calibration Graphs and Detection Limits

Under the optimum condition defined, the calibration graphs for albumin samples were obtained, and the results are shown in Table 3. It can be seen that there is a linear relationship between the fluorescence intensity of the system and the concentration of analytes in the range of 0.2 × 10^−6^–1.3 × 10^−6^ mol L^−1^ for BSA, 0.2 × 10^−6^–1.4 × 10^−6^ mol L^−1^ for HSA standard solutions, 0.2 × 10^−6^–1 × 10^−6^ mol L^−1^ for HSA in plasma samples. The detection limits (S/*N* = 3) for BSA, HSA standard solutions, and plasma samples were 8.7 × 10^−8^ mol L^−1^, 6.2 × 10^−8^ mol L^−1^, and 8.1 × 10^−8^ mol L^−1^, respectively.

#### 3.4.2. Precision and Accuracy

The results of intra-assay precision and accuracy of calibration standards are shown in Table 4. All relative standard deviation (RSD%) was below 5% for standard samples and below 15% for plasma samples which were acceptable for bioanalytical methods according to FDA recommendations [27]. Inter- and intra-assay precisions along with accuracy for quality control samples are listed in Table 5. Similar results obtained for these validation experiments showed that the developed method is both accurate and precise.

#### 3.4.3. Recovery

The recoveries for the investigated albumin samples are summarized in Table 6. The mean recoveries for BSA, HSA, and HSA in plasma were 100.7%, 99.3%, and 95.9%, respectively.

#### 3.4.4. Stability

Under the optimum conditions proposed stability of the probe was investigated. It was found that the fluorescence intensity is stable for about 50 min at room temperature (25°C) after addition of all reagents. Stability at refrigerator temperature (4°C) for three days after addition of all reagents was investigated. After one day decrease in the concentration of analyte is highest but in the second and third days the deviation is less than the first day. The stability results at refrigerator temperature (4°C) are summarized in Table 7.

#### 3.4.5. Analysis of Plasma Samples

The calibration curve in plasma was used for the determination of HSA in the human plasma and compared with the UV spectrophotometric method. The results are shown in Table 8. This method of measuring protein is more sensitive compared with the routine method.

## 4. Conclusions

In this paper, a new fluorimetric method for the determination of albumin is reported. Under optimum conditions, the enhanced intensity of fluorescence is in proportion to the concentration of albumin. Compared with standard spectrophotometric method, the developed method has the advantages of the simplicity and fast processing, low detection limits and wide linear range. So the proposed method is applicable to the determination of albumin in plasma samples in decreased or enhanced levels. The developed method is applicable in bioanalytical laboratories as routine albumin analysis method.

## Figures and Tables

**Table 1:** Effect of the addition order of reagents on the enhanced fluorescence intensity (ΔF%).

Reagent	Reagent	Reagent	Reagent	ΔF%
Tb^3+^	Dano	Albumin	Buffer	46.6
Tb^3+^	Dano	Buffer	Albumin	37.5
Tb^3+^	Buffer	Dano	Albumin	30.9
Tb^3+^	Buffer	Albumin	Dano	36.9
Tb^3+^	Albumin	Dano	Buffer	34.7
Tb^3+^	Albumin	Buffer	Dano	43.5
Dano	Tb^3+^	Albumin	Buffer	39.5
Dano	Tb^3+^	Buffer	Albumin	41.7
Dano	Buffer	Tb^3+^	Albumin	37.3
Dano	Buffer	Albumin	Tb^3+^	44.2
Dano	Albumin	Buffer	Tb^3+^	43.0
Dano	Albumin	Tb^3+^	Buffer	41.3
Buffer	Tb^3+^	Dano	Albumin	26.5
Buffer	Tb^3+^	Albumin	Dano	24.5
Buffer	Albumin	Dano	Tb^3+^	40.7
Buffer	Albumin	Tb^3+^	Dano	33.6
Buffer	Dano	Albumin	Tb^3+^	41.7
Buffer	Dano	Tb^3+^	Albumin	40.1
Albumin	Tb^3+^	Dano	Buffer	37.6
Albumin	Tb^3+^	Buffer	Dano	40.1
Albumin	Dano	Buffer	Tb^3+^	39.5
Albumin	Dano	Tb^3+^	Buffer	43.1
Albumin	Buffer	Tb^3+^	Dano	31.9
Albumin	Buffer	Dano	Tb^3+^	25.8

Analytical conditions: [Tb^3+^]: 8.5 × 10^−5^ mol L^−1^; [Dano]: 1.5 × 10^−4^ mol L^−1^; [HSA]: 1 × 10^−6^ mol L^−1^; pH = 7.8.

**Table 2:** Interference effect on the enhanced fluorescence intensity(ΔF%).

Interfere substance	Concentration of interfere substance (×10^−3^ mol L^−1^)	Tolerance ratio (species/HSA)	Chang of ΔI_F_%
Na^+^(Cl^−^)	5	5000	1.3
Fe^3+^ (Cl^−^)	0.005	5	−4.2
K^+^ (CL^−^)	3	3000	−2.3
NH_4_^+^ (Cl^−^)	4.2	4200	−4.7
Saccharose	1	1000	3.7
Glycine	1	1000	−2.4
Mn^2+^ (Cl^−^)	0.05	50	−3.8
ctDNA	0.00075	0.75	3.0
Al^3+^ (Cl^−^)	0.005	5	−4.9
Ba^2+^ (Cl^−^)	1.4	1400	−3.4
Ca^2+^ (Cl^−^)	1.15	1150	−4.2
Na^+^ (CO_3_^2−^)	1.19	1900	−3.7

Analytical conditions: [Tb^3+^]: 8.5 × 10^−5^ mol L^−1^; [Dano]: 1.5 × 10^−4^ mol L^−1^; [HSA]: 1 × 10^−6^ mol L^−1^; pH = 7.8.

**Table 3:** Validation data of the proposed method for quantification of BSA, HSA, and HSA in human plasma.

Parameters	BSA	HSA	HSA in plasma
Linear range (mol L^−1^)	0.2 × 10^−6^–1.3 × 10^−6^	0.2 × 10^−6^–1.4 × 10^−6^	0.2 × 10^−6^–1 × 10^−6^
Slope	32.775	42.574	217.130
Intercept	3.9446	0.2742	−14.6410
Correlation coefficient	0.9991	0.9990	0.9899
Number of data points	12	13	9
LOD (mol L^−1^)	8.7 × 10^−8^	6.2 × 10^−8^	8.1 × 10^−8^

**Table 4:** Intra-assay precision and accuracy of calibration standards.

Analyte name	Nominal concentration (mol L^−1^) (*N* = 3)	Found concentration (mol L^−1^) (*N* = 3)	Precision (RSD%)	Accuracy (RE %)
BSA	0.3 × 10^−6^	0.286 × 10^−6^	2.78	−4.5
	0.5 × 10^−6^	0.489 × 10^−6^	3.91	−2.2
	0.7 × 10^−6^	0.714 × 10^−6^	3.97	1.9
	1.0 × 10^−6^	0.992 × 10^−6^	0.50	−0.8
	1.2 × 10^−6^	1.194 × 10^−6^	2.78	−0.5

HSA	0.3 × 10^−6^	0.308 × 10^−6^	2.85	2.9
	0.5 × 10^−6^	0.510 × 10^−6^	3.19	2.1
	0.7 × 10^−6^	0.713 × 10^−6^	3.14	1.9
	1.0 × 10^−6^	0.994 × 10^−6^	1.53	−0.5
	1.2 × 10^−6^	1.185 × 10^−6^	1.44	−1.2

HSA in plasma	0.3 × 10^−6^	0.277 × 10^−6^	4.13	−7.6
	0.5 × 10^−6^	0.519 × 10^−6^	3.94	3.7
	0.7 × 10^−6^	0.711 × 10^−6^	5.08	1.6
	0.8 × 10^−6^	0.791 × 10^−6^	4.23	−1.0
	1.0 × 10^−6^	0.959 × 10^−6^	3.58	−4.1

**Table 5:** Assay precision and accuracy of quality control samples.

Analyte name	Concentration (mol L^−1^)	Intra-assay precision (RSD%)	Inter-assay precision (RSD%)	Accuracy (RE%)
BSA	0.3 × 10^−6^	2.62	4.55	−3.9
	0.7 × 10^−6^	4.39	1.70	1.1
	1.2 × 10^−6^	4.70	3.57	2.6

HSA	0.3 × 10^−6^	3.19	4.07	4.4
	0.7 × 10^−6^	2.79	4.62	3.1
	1.2 × 10^−6^	2.00	2.60	−0.4

HSA in plasma	0.3 × 10^−6^	6.44	8.08	−6.3
	0.5 × 10^−6^	4.16	4.90	1.5
	0.8 × 10^−6^	3.85	3.58	−2.7

**Table 6:** Absolute and mean recoveries for the studied analytes.

Analyte name	Nominal concentration (mol L^−1^) (*N* = 5)	Found concentration (mol L^−1^) (*N* = 5)	Recovery %	Mean recovery %	Precision of recovery (RE%)
BSA	0.3 × 10^−6^	0.297 × 10^−6^	99	100.7	−1.0
	0.7 × 10^−6^	0.698 × 10^−6^	99.7		−0.3
	1.2 × 10^−6^	1.240 × 10^−6^	103.3		3.3

HSA	0.3 × 10^−6^	0.294 × 10^−6^	98	99.3	−2.0
	0.7 × 10^−6^	0.705 × 10^−6^	100.7		0.70
	1.2 × 10^−6^	1.190 × 10^−6^	99.2		−0.8

HSA in plasma	0.3 × 10^−6^	0.283 × 10^−6^	94.3	95.9	−5.7
	0.5 × 10^−6^	0.489 × 10^−6^	97.8		−2.2
	0.8 × 10^−6^	0.765 × 10^−6^	95.6		−4.4

**Table 7:** Stability at refrigerator (4°C) for three days.

Analyte	Concentration (mol L^−1^)	After one day at 4°C		After two days at 4°C		After three days at 4°C	
		Concentration found (mol L^−1^)	Accuracy (RE%)	Concentration found (mol L^−1^)	Accuracy (RE%)	Concentration found (mol L^−1^)	Accuracy (RE%)
BSA	0.3 × 10^−6^	0.224 × 10^−6^	−25.3	0.216 × 10^−6^	−28.1	0.210 × 10^−6^	−29.9
	0.7 × 10^−6^	0.624 × 10^−6^	−10.8	0.606 × 10^−6^	−13.4	0.589 × 10^−6^	−15.7
	1.2 × 10^−6^	1.019 × 10^−6^	−15	0.983 × 10^−6^	−18.1	0.962 × 10^−6^	−19.8

HSA	0.3 × 10^−6^	0.26 × 10^−6^	−14.1	0.251 × 10^−6^	−16.1	0.249 × 10^−6^	−16.8
	0.7 × 10^−6^	0.583 × 10^−6^	−16.6	0.569 × 10^−6^	−18.7	0.565 × 10^−6^	−19.2
	1.2 × 10^−6^	0.995 × 10^−6^	−17.1	0.982 × 10^−6^	−18.2	0.945 × 10^−6^	−21.2

HSA in plasma	0.3 × 10^−6^	0.217 × 10^−6^	−27.5	0.216 × 10^−6^	−27.9	0.214 × 10^−6^	−28.8
	0.5 × 10^−6^	0.430 × 10^−6^	−13.9	0.421 × 10^−6^	−15.8	0.415 × 10^−6^	−16.9
	0.8 × 10^−6^	0.649 × 10^−6^	−18.9	0.641 × 10^−6^	−19.8	0.637 × 10^−6^	−20.4

**Table 8:** Determinations of HSA in different plasma samples obtained from different human blood samples.

Sample number	The proposed method	Standard UV spectrophotometry
	Found concentration (×10^−4 ^mol L^−1^) (RSD%) (*N* = 3)	Found concentration (×10^−4 ^mol L^−1^) (RSD%) (*N* = 3)
1	6.93	6.78
	(4.5)	(1.2)

2	6.36	6.11
	(3.4)	(2.8)

3	6.46	6.20
	(8.6)	(3.9)

4	6.35	6.25
	(4.1)	(2.2)

5	6.40	6.26
	(5.2)	(2.4)

6	6.09	6.01
	(9.4)	(1.4)

7	6.87	6.69
	(8.7)	(2.1)
